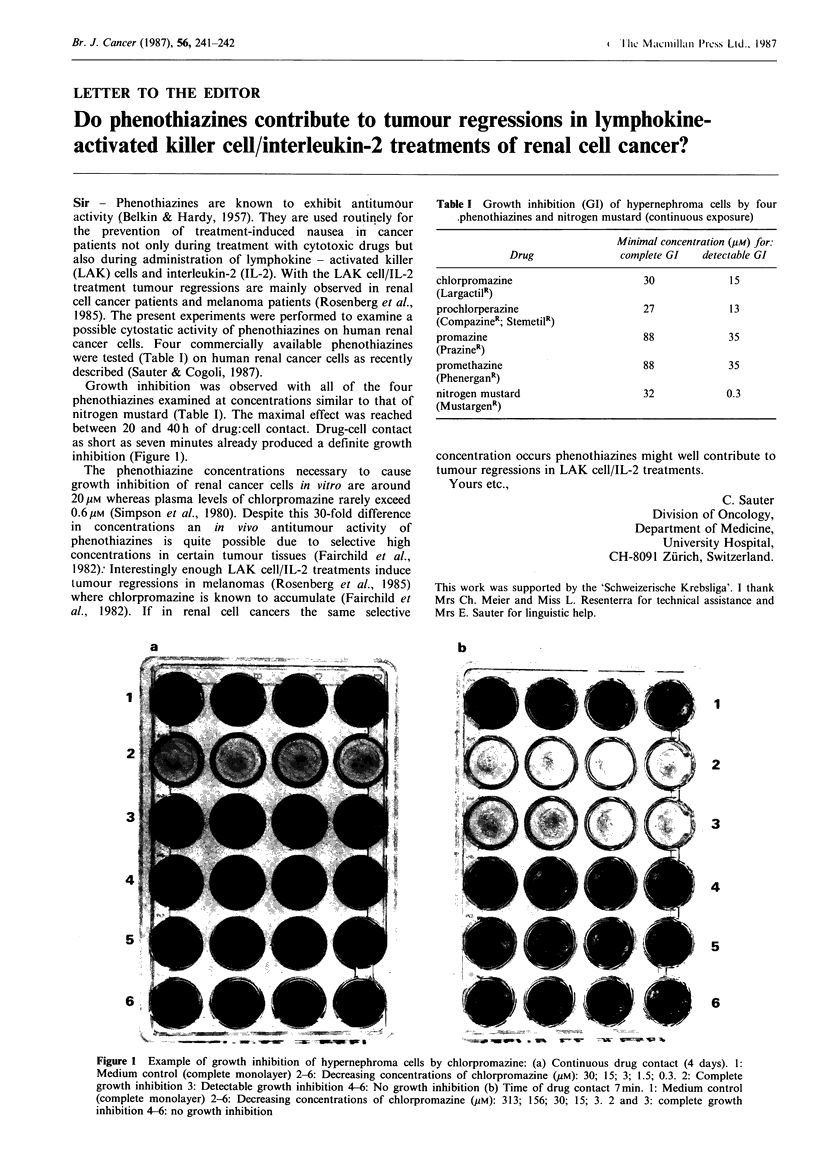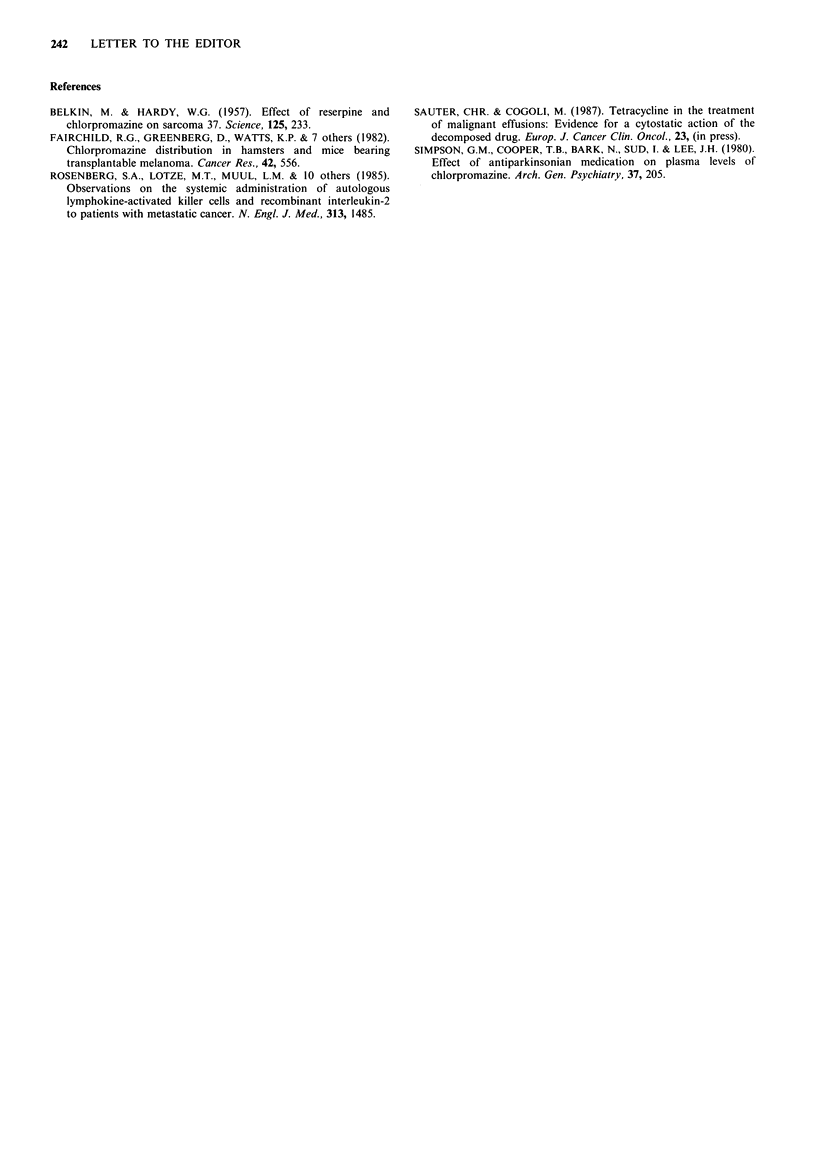# Do phenothiazines contribute to tumour regressions in lymphokine-activated killer cell/interleukin-2 treatments of renal cell cancer?

**DOI:** 10.1038/bjc.1987.182

**Published:** 1987-08

**Authors:** C. Sauter

## Abstract

**Images:**


*Br. J. Cancer* (1987), **56**, 241–242 © The Macmillan Press Ltd., 1987

LETTER TO THE EDITOR

### Do phenothiazines contribute to tumour regressions in lymphokine-activated killer cell/interleukin-2 treatments of renal cell cancer?

**Sir** – Phenothiazines are known to exhibit antitumour activity (Belkin & Hardy, 1957). They are used routinely for the prevention of treatment-induced nausea in cancer patients not only during treatment with cytotoxic drugs but also during administration of lymphokine – activated killer (LAK) cells and interleukin-2 (IL-2). With the LAK cell/IL-2 treatment tumour regressions are mainly observed in renal cell cancer patients and melanoma patients (Rosenberg *et al.*, 1985). The present experiments were performed to examine a possible cytostatic activity of phenothiazines on human renal cancer cells. Four commercially available phenothiazines were tested (Table I) on human renal cancer cells as recently described (Sauter & Cogoli, 1987).

Growth inhibition was observed with all of the four phenothiazines examined at concentrations similar to that of nitrogen mustard (Table I). The maximal effect was reached between 20 and 40 h of drug:cell contact. Drug-cell contact as short as seven minutes already produced a definite growth inhibition (Figure 1).

The phenothiazine concentrations necessary to cause growth inhibition of renal cancer cells *in vitro* are around 20 μM whereas plasma levels of chlorpromazine rarely exceed 0.6 μM (Simpson *et al.*, 1980). Despite this 30-fold difference in concentrations an *in vivo* antitumour activity of phenothiazines is quite possible due to selective high concentrations in certain tumour tissues (Fairchild *et al.*, 1982). Interestingly enough LAK cell/IL-2 treatments induce tumour regressions in melanomas (Rosenberg *et al.*, 1985) where chlorpromazine is known to accumulate (Fairchild *et al.*, 1982). If in renal cell cancers the same selective concentration occurs phenothiazines might well contribute to tumour regressions in LAK cell/IL-2 treatments.

**Table I** Growth inhibition (GI) of hypernephroma cells by four phenothiazines and nitrogen mustard (continuous exposure)

| Drug | Minimal concentration (μM) for: complete GI | detectable GI |
|---|---|---|
| chlorpromazine (Largactil$^R$) | 30 | 15 |
| prochlorperazine (Compazine$^R$; Stemetil$^R$) | 27 | 13 |
| promazine (Prazine$^R$) | 88 | 35 |
| promethazine (Phenergan$^R$) | 88 | 35 |
| nitrogen mustard (Mustargen$^R$) | 32 | 0.3 |

Yours etc.,

C. Sauter
Division of Oncology,
Department of Medicine,
University Hospital,
CH-8091 Zürich, Switzerland.

This work was supported by the 'Schweizerische Krebsliga'. I thank Mrs Ch. Meier and Miss L. Resenterra for technical assistance and Mrs E. Sauter for linguistic help.

**Figure 1** Example of growth inhibition of hypernephroma cells by chlorpromazine: (a) Continuous drug contact (4 days). 1: Medium control (complete monolayer) 2–6: Decreasing concentrations of chlorpromazine (μM): 30; 15; 3; 1.5; 0.3. 2: Complete growth inhibition 3: Detectable growth inhibition 4–6: No growth inhibition (b) Time of drug contact 7 min. 1: Medium control (complete monolayer) 2–6: Decreasing concentrations of chlorpromazine (μM): 313; 156; 30; 15; 3. 2 and 3: complete growth inhibition 4–6: no growth inhibition

### References

BELKIN, M. & HARDY, W.G. (1957). Effect of reserpine and chlorpromazine on sarcoma 37. *Science*, **125**, 233.

FAIRCHILD, R.G., GREENBERG, D., WATTS, K.P. & 7 others (1982). Chlorpromazine distribution in hamsters and mice bearing transplantable melanoma. *Cancer Res.*, **42**, 556.

ROSENBERG, S.A., LOTZE, M.T., MUUL, L.M. & 10 others (1985). Observations on the systemic administration of autologous lymphokine-activated killer cells and recombinant interleukin-2 to patients with metastatic cancer. *N. Engl. J. Med.*, **313**, 1485.

SAUTER, CHR. & COGOLI, M. (1987). Tetracycline in the treatment of malignant effusions: Evidence for a cytostatic action of the decomposed drug. *Europ. J. Cancer Clin. Oncol.*, **23**, (in press).

SIMPSON, G.M., COOPER, T.B., BARK, N., SUD, I. & LEE, J.H. (1980). Effect of antiparkinsonian medication on plasma levels of chlorpromazine. *Arch. Gen. Psychiatry*, **37**, 205.